# Chronic kidney disease mineral bone disorder in childhood and young adulthood: a ‘growing’ understanding

**DOI:** 10.1007/s00467-023-06109-3

**Published:** 2023-08-25

**Authors:** Alexander D. Lalayiannis, Emilia M. D. Soeiro, Rosa M. A. Moysés, Rukshana Shroff

**Affiliations:** 1 Birmingham Women’s and Children’s NHS Foundation Trust, Birmingham, UK; 2 University College London Great Ormond Street Hospital Institute of Child Health, London, UK; 3 Universidade Federal de Pernambuco, Recife, Brazil; 4 Sao Paulo University Faculty of Medicine, Universidade de Sao Paulo Faculdade de Medicina, São Paulo, Brazil

**Keywords:** Chronic kidney disease mineral and bone disorder, Children, Growth, Bone turnover, Mineralization, Vascular calcification

## Abstract

Chronic kidney disease (CKD) mineral and bone disorder (MBD) comprises a triad of biochemical abnormalities (of calcium, phosphate, parathyroid hormone and vitamin D), bone abnormalities (turnover, mineralization and growth) and extra-skeletal calcification. Mineral dysregulation leads to bone demineralization causing bone pain and an increased fracture risk compared to healthy peers. Vascular calcification, with hydroxyapatite deposition in the vessel wall, is a part of the CKD-MBD spectrum and, in turn, leads to vascular stiffness, left ventricular hypertrophy and a very high cardiovascular mortality risk. While the growing bone requires calcium, excess calcium can deposit in the vessels, such that the intake of calcium, calcium- containing medications and high calcium dialysate need to be carefully regulated. Normal physiological bone mineralization continues into the third decade of life, many years beyond the rapid growth in childhood and adolescence, implying that skeletal calcium requirements are much higher in younger people compared to the elderly. Much of the research into the link between bone (de)mineralization and vascular calcification in CKD has been performed in older adults and these data must not be extrapolated to children or younger adults. In this article, we explore the physiological changes in bone turnover and mineralization in children and young adults, the pathophysiology of mineral bone disease in CKD and a potential link between bone demineralization and vascular calcification.

**Graphical abstract:**

**Figure:**
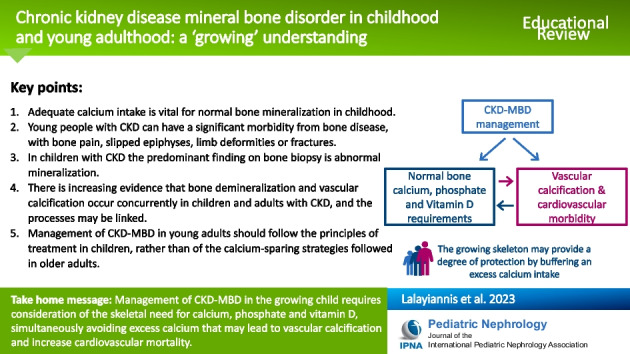
A higher resolution version of the Graphical abstract is available as Supplementary information

**Supplementary Information:**

The online version contains supplementary material available at 10.1007/s00467-023-06109-3.

## Introduction

An important component of chronic kidney disease (CKD) is mineral and bone disorder (CKD-MBD). Kidney Disease Improving Global Outcomes (KDIGO) defines MBD as the triad of biochemical abnormalities (calcium, phosphate, parathyroid hormone and vitamin D), bone abnormalities (turnover, mineralization, volume and growth) and extra-skeletal calcification [1]. Bone demineralization caused by mineral homeostatic imbalance, is a key factor in the increased bone-related morbidity, fracture risk, cardiovascular disease (CVD), and mortality seen in CKD-MBD [1, 2].

The skeletal requirements for calcium differ at different stages of physiological growth and skeletal maturation. The growing bone must avidly absorb calcium in order to mineralize, and calcium requirements are highest during periods of rapid growth such as infancy and adolescence. Thereafter, under physiological conditions, skeletal mineralization must continue until the third decade of life, albeit at a much slower rate, before stabilizing and potentially demineralizing in late adulthood. The calcium requirements of the skeleton help determine the calcium balance of the individual. The MBD of CKD significantly impacts on normal bone turnover and mineralization processes leading to reduced bone strength, bone pain, fractures, and short stature. Moreover, when the normal bone calcium uptake is impaired, excess calcium may be deposited in soft tissues, including the vasculature.

In this article we explore the physiological changes in the skeleton from childhood to adulthood, the normal skeletal mineralization process, and the effects of CKD on bone health. We also examine a possible link between bone demineralization and vascular calcification through imaging and biomarker studies. We stress the importance of approaching CKD-MBD in children and young adults as a continuum, and not extrapolating data from studies in older adults with CKD to younger patients.

## Normal bone turnover and mineralization in childhood and adulthood

The normal bone undergoes a constant cycle of modelling and remodelling. During modelling new bone is formed and minerals are deposited. In childhood and adolescence modelling is the predominant process that leads to the bones changing shape and elongating. The periosteum expands alongside longitudinal growth, with more minerals accrued and laid down. Remodelling involves resorption of old bone and replacement with new bone through formation. Modelling predominates in childhood and early adult life as the bone grows and gains strength, and therefore, mineral accrual is at its highest. In later adulthood, remodelling is the predominant process. Mineral consumption is in a neutral balance after the mid-30 s until older age, when physiological bone demineralization tends to occur [3].

The process of bone formation and resorption is collectively known as bone turnover and reflects the continuous metabolic activity of the skeleton. The two bone compartments, the metabolically active inner trabecular compartment and the mineral rich, dense outer cortical layer exist together to provide the mechanical function of anchoring muscles and tendons, allowing mobility, and maintaining body structure and posture. If there is any imbalance in the resorption/formation process towards resorption this can lead to a porous cortical compartment and thinning of the trabeculae resulting in demineralization and osteoporosis [4].

**Fig. 1:**
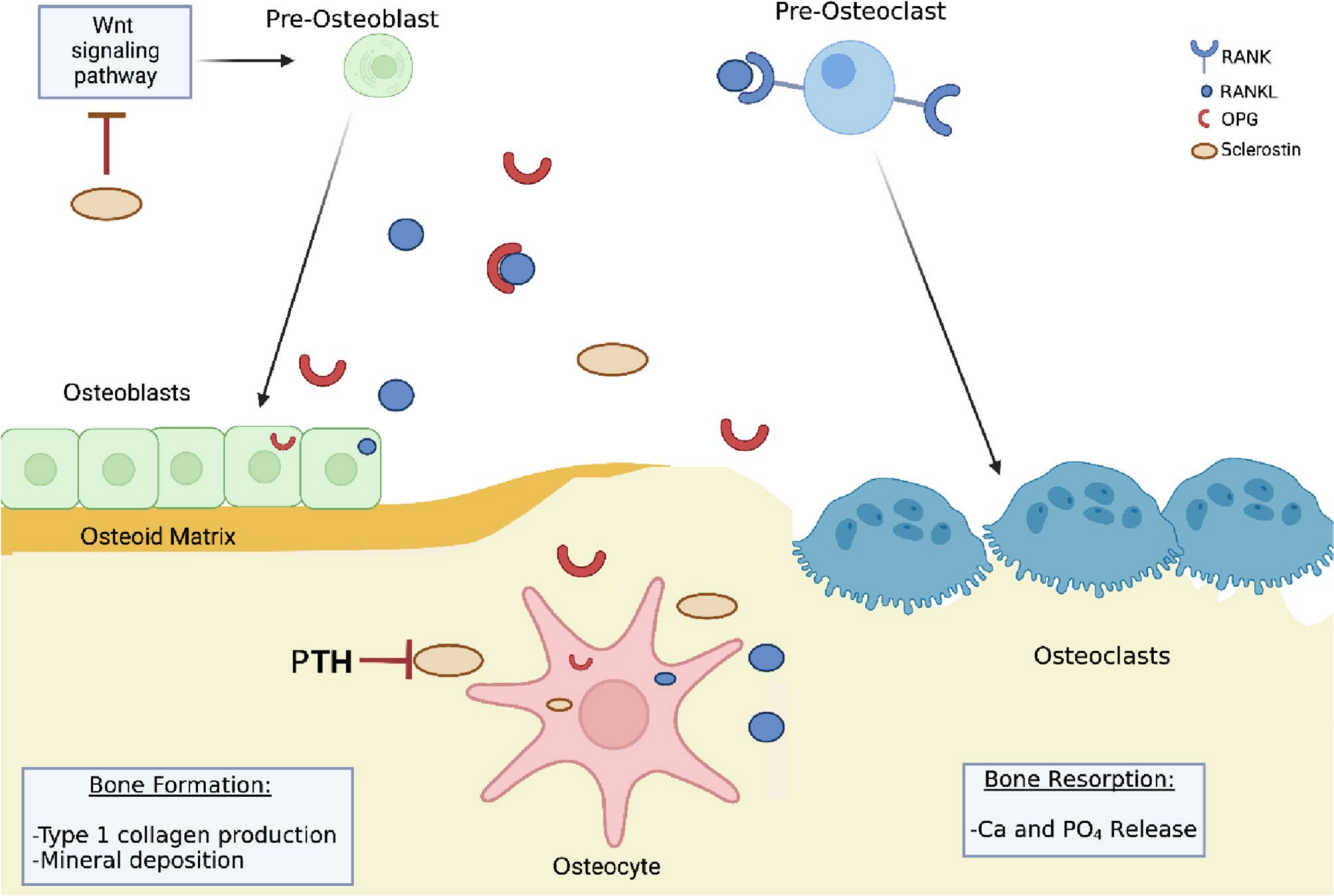
A schematic diagram of the normal bone mineralization process. Osteoblasts produce type 1 collagen which becomes the scaffolding around which mineralization occurs to form the osteoid. Osteoblastic differentiation is mainly regulated by the Wnt signalling pathway. Hormones such as PTH and vitamin D regulate this process. Secretory calcium-binding phosphoproteins (SCPPs), such as osteonectin, bind to hydroxyapatite and Type 1 collagen. Other SCPPs are osteopontin and bone sialoprotein which are used as a focus for mineral crystal formation. Osteoclasts control bone resorption. Mature osteoclasts bind to bone matrix and secrete lysozymal enzymes to then release calcium and phosphate into the serum. Resorption is activated by the RANK–RANKL–OPG pathway. Osteoclast precursors express RANK, which is activated by its ligand, RANKL, produced by osteoblasts and osteocytes. Osteoprotegerin (OPG), also a product of osteoblasts and osteocytes, is a competitor receptor for RANKL with the opposite effect of neutralising and pausing the osteoclastic function activated by the RANKL–RANK complex. Osteocytes are terminally differentiated osteoblasts, embedded in mineralised osteoid. Osteocytes upregulate osteoblasts through nitric oxide and prostaglandin E2 production or downregulate them through sclerostin secretion

The modelling–remodeling cycle is controlled by three cell types: osteoblasts on the bone surface that deposit new bone matrix; osteocytes embedded in bone that are terminally differentiated from osteoblasts and function as mechanical and metabolic sensors; and the mineralized osteoid-resorbing osteoclasts (See Fig. 1).

### Osteoblasts

Osteoblasts are the primary cells that form bone. They produce type 1 collagen, which becomes the scaffolding around which mineralization occurs to form the osteoid. Phosphates from matrix vesicles combine with calcium and other elements to form hydroxyapatite crystals that are deposited around the preformed collagen scaffold in a controlled process. Osteoblastic differentiation is mainly regulated by the canonical Wnt signalling pathway that plays a central role in normal bone development and homeostasis, as well as bone repair and regeneration following injury [5]. The Wnt-β-catenin pathway regulates the differentiation of pluripotent mesenchymal stem cells into either osteoblasts or chondrocytes, and in the later stages of repair, form pre-osteoblasts to differentiate into osteoblasts.

### Osteoclasts

Osteoclasts are responsible for controlling bone resorption. Mature osteoclasts bind to bone matrix, becoming polarised. The side of the cell in contact with the area to be resorbed is called the ruffled border and this secretes metalloproteinases, lysozymes and cathepsin K that drive resorption. The collagen, calcium and phosphate that result from this process are in turn endocytosed by the osteoclast, with calcium and phosphate released into the blood [6]. Resorption is activated by the RANK–RANKL–OPG pathway, activating osteoclasts and driving their differentiation. Osteoclast precursors express RANK (receptor activator of nuclear factor-κß), which is activated by its ligand, RANKL, produced by osteoblasts and osteocytes. Osteoprotegerin (OPG), also a product of osteoblasts and osteocytes, is a decoy receptor for RANKL with a high affinity for the RANK receptor, but with the opposite effect of neutralising and pausing the osteoclastic function activated by the RANKL–RANK complex. Thus, the RANKL/OPG ratio is an important determinant of bone mass.

### Osteocytes

As the bone matrix is deposited by osteoblasts, some of these cells undergo terminal differentiation into osteocytes and become embedded in the lacunae in the mineralized osteoid [7, 8]. Eventually, osteocytes become the most common cell type in bone, comprising more than 90% of bone cells. Their long dendritic processes allow them to connect and communicate with each other and detect the mechanical forces placed upon the skeleton as well as detect microdamage [3]. Osteocytes in turn upregulate osteoblasts through nitric oxide and prostaglandin E2 production or downregulate them through sclerostin secretion. Sclerostin binds to growth factors, preventing osteoblast differentiation and also prevents the activation of the Wnt signalling pathway [9]. Osteocytes also produce Fibroblast Growth Factor-23 (FGF23), which together with its co-receptor Klotho, induces phosphaturia [10, 11] and suppresses calcitriol production [12].

### Mineralization

The mineral component of bone is hydroxyapatite [Ca_10_(PO_4_)_6_(OH)_2_] as well as crystals of calcium and phosphate coupled with carbonate, sodium and magnesium [13]. Osteoblasts promote bone mineralization by depositing the protein component of the extracellular bone matrix, and mineralization follows. Hormones such as PTH and vitamin D, as well as secretory calcium-binding phosphoproteins (SCPPs) expressed by osteoblasts regulate this process. SCPP proteins, such as osteonectin, bind to hydroxyapatite and type 1 collagen. Other SCPPs are osteopontin and bone sialoprotein which provide a focus for mineral crystal formation. The extent of bone mineralization is dependent on calcium availability, but also on the extracellular levels of phosphate and pyrophosphate (P_2_O_7_^4−^, a phosphate-containing inhibitor of hydroxyapatite crystal growth and thus of mineralization) controlled by alkaline phosphatase enzymes. Bone alkaline phosphatase may increase local phosphorus concentrations, remove pyrophosphate, or modify phosphoproteins to control their ability to act as facilitators of crystallization [13].

## Bone growth

During the growth period, the modelling process at the long bones occurs at the growth plate. In this area, new bone is built in places that were previously occupied by cartilage. Flat chondrocytes become hypertrophied and then become part of the mineralizing cartilage. These cells are arranged in columns that line up together, simultaneously. As the chondrocytes at the bottom become hypertrophic, and are penetrated by osteoblasts that form spongy bone, there is a proliferation of the flat chondrocytes at the top of the growth plate. This process is controlled by several factors, including the role of Indian hedgehog (Ihh) and parathyroid hormone-related protein (PTHrP) [14]. There is a negative-feedback loop, where PTHrP is secreted from perichondrial cells and chondrocytes at the ends of long bones and acts on receptors of proliferating chondrocytes to ensure continued proliferation. Conversely, when PTHrP production decreases or is sufficiently distant, Ihh is produced, increasing the rate of proliferation and stimulating PTHrP synthesis at the ends of bones [15]. However, PTHrP and PTH share the same receptor [16]. This may be a cause for the growth defects that are commonly seen in children with CKD, if PTH levels are persistently high. Long term exposure to high PTH levels leads to fibrous changes in the bones, and these may play a role in hindering bone growth [17].

## Role of vitamin D in bone mineralization

Vitamin D is an important regulator of bone health, both by increasing calcium and phosphate absorption from the gut and by regulating bone mineralization. Vitamin D deficiency leads to decreased calcium absorption and ultimately the release of calcium from the bones in order to maintain circulating calcium concentrations [18], resulting in poor bone mineralization and rickets in children and osteoporosis in adults.

Vitamin D is a key player in bone mineralization, not solely through adequate intestinal calcium absorption, but also through the action of active 1,25-dihydroxyvitamin D [1,25(OH)_2_D] in growth plate development, optimal osteoblastic bone formation and bone resorption [19]. Although there is some conflicting literature, as some studies have shown inhibition of osteoclastogenesis by 1,25(OH)_2_D in CKD models and controls [20], it is thought that 1,25(OH)_2_D stimulates osteoclastogenesis, by upregulating expression of the RANK ligand by osteoblasts and the RANK-ligand receptor on osteoclast precursor cells [21]. The RANK receptor binds RANKL and induces maturation of preosteoclasts to osteoclasts. Osteoclasts then increase the resorptive potential of the bone, releasing calcium into the blood [22]. Whilst this calcium release from the bone is accompanied by phosphate release as well, FGF-23 secreted by osteocytes works to increase phosphaturia in the distal tubule by upregulating sodium-phosphate transporters to prevent hyperphosphatemia [23], at least in healthy individuals and those with early stages of CKD [11].

Although the overall benefits of Vitamin D are debated [24], it is recognised that adequate Vitamin D levels prevent nutritional rickets, suppress PTH [25–27], and increase intestinal calcium absorption [28]. There is some indication that Vitamin D repletion is associated with improved lumbar spine BMD [29]. In health and in CKD, Vitamin D is crucial in the mineralization of bone matrix and thus bone formation.

## Normal calcium requirements in healthy individuals of different ages

Calcium in sufficient quantities is required during the rapid growth phase of childhood and adolescence, in order to mineralize newly formed bone. An adequate intake and absorption of calcium, together with genetic influences, physical activity, nutrition and lifestyle factors is required in order to promote skeletal growth and mineralization [30]. The skeletal mass increases from a mere 25 g at birth to around 1000–1200 g in adult males and females. Thus, children, particularly in infancy and adolescence, have a higher demand for calcium compared to adults, and are in positive calcium balance [31]. A review of 519 calcium balance studies performed on participants from birth to 30 years old showed that calcium balance correlated positively with oral calcium intake. The highest calcium requirement was in the first year of life (503 ± 91 mg/day) and during pubertal growth (396 ± 164 mg/day). Adult requirements were considerably lower thereafter (114 ± 133 mg/day) (see Table 1) [31].

**Table 1:** Threshold intake and mean calcium balance per age group

Age group	Threshold intake (mg/day)	Threshold balance (mg/day)
0–1 yrs	1090	+ 503 ± 91
2–8 yrs	1390	+ 246 ± 126
9–17 yrs	1480	+ 396 ± 164
18–30 yrs	957	+ 114 ± 133
> 30 yrs	583	+ 72 ± 35

Following analysis of calcium balance studies in 519 healthy individuals, this table shows the threshold intake at which the calcium balance does not increase further with increasing intake, and the mean balance for each age group. Under the age of 30 years, the calcium balance is always positive due to skeletal calcium accrual, but as more mineral is laid down, and peak bone mass is reached, the balance mean becomes smaller. Adapted from Matkovic & Heaney [26]. Over 30 yrs adapted from [32–34]

A higher calcium intake in the diet has a direct effect on bone mineral density (BMD) in healthy children [35–37]. A daily consumption of a high calcium intake was associated with a greater increase in radial and femoral BMD in healthy pre-pubescent girls [38], and a significant increase in BMD (9.6% vs. 8.5%, *P* = 0.017) and bone mineral content (27.0% vs. 24.1%, *P* = 0.009) by dual energy x-ray absorptiometry (DXA) in 12-year-old girls studied over an 18-month period [39]. A randomized trial of 354 adolescent girls showed that increased calcium intake was associated with a significantly higher radial and total body BMD on DXA scan over the 7-year follow up [40].

**Fig. 2:**
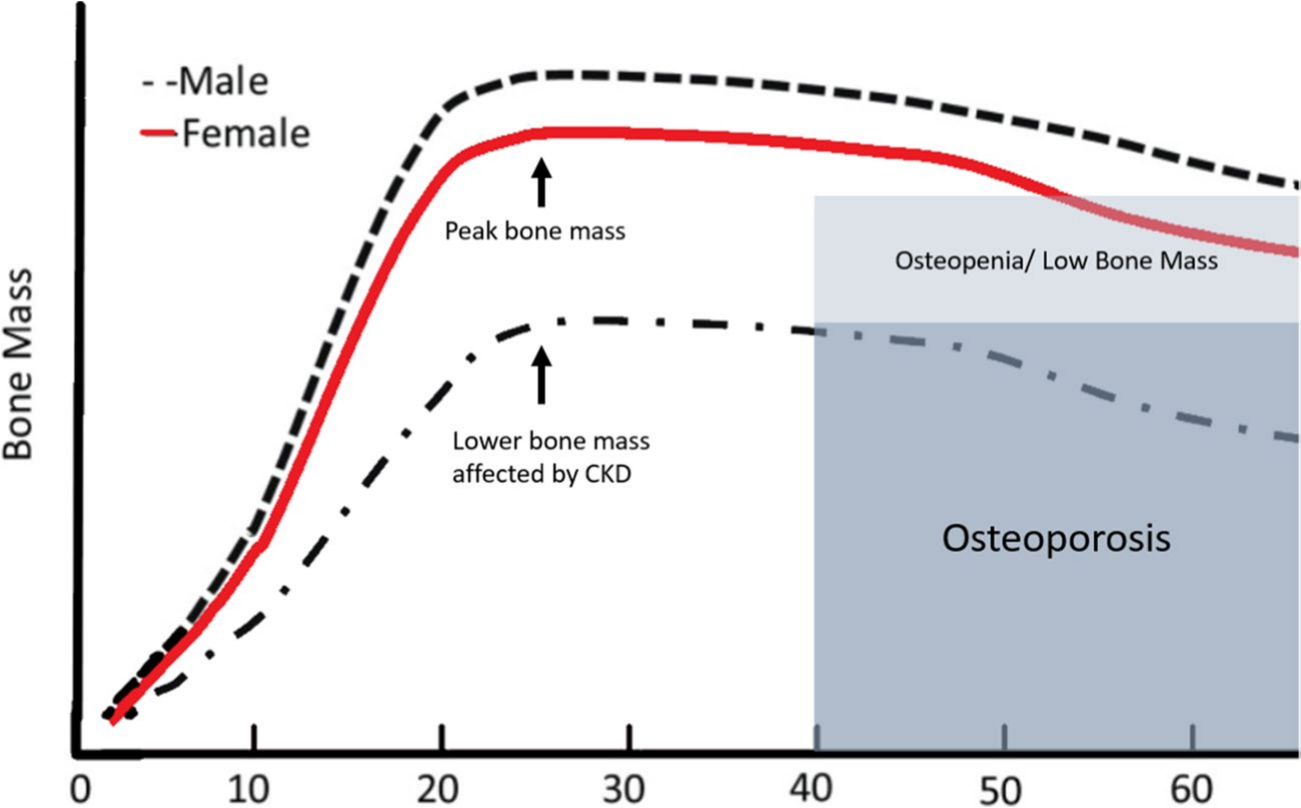
Peak bone mass and the risk of osteoporosis with CKD. Bone mass attainment, through sufficient mineral accrual in childhood, adolescence and young adulthood is crucial in preventing lower bone mineral density in later life. Adapted from Weaver et al. 2016 [27], with permission

When bone growth ceases, bone elongation has reached its maximum, and mineralization only occurs at the sites of bone remodelling and the adult requirements for calcium are reduced. This bone mineralization threshold, also known as ‘peak bone mass’ (PBM), reflects the maximum mineral content in bone [35] (Fig. 2). Cross-sectional and longitudinal studies have suggested that PBM is achieved in the third decade of life. In 156 healthy adult women, the mineral accrual stopped by 28.3 to 29.5 years of age [41]. In 300 healthy females aged 6 to 32 years, bone mineral content was highest in the early twenties (23.0 ± 1.4 years); but increased into the early thirties [42]. Studies looking at dietary information with serial DXA scans longitudinally from childhood to early thirties showed that peak bone mass was achieved early in the third decade of life, many years after peak height velocity of bone growth had been reached. Thus, skeletal mineralization continues long after the genetically predetermined height potential is reached [43].

The US National Osteoporosis Foundation position statement on PBM development sets out the available evidence for calcium intake on bone accretion, especially during late childhood and the peripubertal years [35]. This evidence also shows that bone mass attainment from childhood to young adulthood is crucial in preventing osteoporosis and osteopenia later in life [35].

## Dysregulated mineral metabolism in CKD

In CKD, the normally tightly regulated calcium–phosphate–PTH–vitamin D axis is disrupted. In early CKD, an increased production of FGF23, together with its co-receptor Klotho, occurs in response to higher levels of serum phosphorous to induce phosphaturia [10, 11] and suppress calcitriol production [12], thereby returning serum phosphorous to normal levels. This early rise of FGF23 precedes the PTH rise [44–46]. Following this, a combination of reduced phosphate excretion and a decrease in 1-alpha-hydroxylation of 25-hydroxyvitamin D by the kidney tissue leads to hypocalcaemia, which in turn leads to increased production of PTH [47]. The hyperparathyroidism aims to keep the serum calcium levels within the normal physiological range by increasing bone resorption and thus calcium release from the bone. In bone, PTH binds to the PTH receptor on the surface of osteoblasts, increasing the expression of RANKL which leads to osteoclast activation. That stimulates resorption which releases calcium, aiming to maintain normal serum levels. This affects the total bone mineral content and increases cortical porosity [48–50]. The PTH effect on bone is also, to some degree, mediated by inhibiting the expression of sclerostin by osteocytes [51]. Sclerostin is secreted by osteocytes to inhibit the differentiation of precursor cells into osteoblasts [52]. It also inhibits the Wnt pathway signalling, acting on osteoblasts to reduce bone formation [53]. Higher levels are found in CKD patients [54] and associated with the reduction in bone formation, but its precise effect on the calcification of vessels has not been fully elucidated [55]. FGF23, also secreted by osteocytes, directly inhibits Wnt signalling pathways which are needed in bone mineralization [56]. Overall, this hyperparathyroid state of increased resorption leads to demineralization, loss of normal bone architecture and an increased fracture risk [57, 58].

As the pathophysiology of CKD-MBD is better understood, the role of biomarkers becomes clearer. Routinely used serum biomarkers such as PTH, calcium, phosphate and alkaline phosphatase are only moderate predictors of the bone turnover, volume and mineralization as assessed by bone biopsy [59]. Their trends over time are used for the best clinical estimation of bone health [60]. A cross-sectional study examining cortical BMD by peripheral quantitative CT (pqCT) scan in children and young adults with CKD or on dialysis, found that serum biomarkers accounted for only 57% of the variability [61]. Lumbar spine DXA was not predictive of cortical BMD as assessed by pQCT [61].

## Clinical and radiological correlations with mineralization defects in CKD

In childhood, cortical demineralization and disrupted bone architecture affect bone formation and growth, with bone pain, limb deformities and poor final height attainment all being described in children with CKD [62–66]. This process starts in the early stages of CKD and worsens as CKD progresses [63, 67]. Severe bone disease can present clinically as slipped epiphyses, abnormal gait, reduced mobility, genu valgum and genu varum [68]. In a cohort of 900 children and adolescents on peritoneal dialysis, limb deformities, pain, and radiological signs of bone disease were present in 15% [66]. The CKD in Children (CKiD) study evaluated the prevalence of fractures in children with CKD. In this young cohort (median age of 11 (7.4–14.5) years) with predominantly mild to moderate CKD (median eGFR 46.5 (34.4–58.5) ml/min/1.73 m^2^), the reported fracture rates were 2.4 and threefold higher in males and females respectively compared to their healthy peers [48] and exceeded those reported in adult haemodialysis patients [69]. The factors independently associated with a higher fracture rate were baseline difficulty in walking, Tanner puberty stages 4–5, greater height Z-score, higher PTH levels, and team sports participation (in ≥ 1 sport: HR 2.35, 95% CI 1.01–5.47, *p* = 0.047; in ≥ 2 sports: HR 4.87, 95% CI 2.21–10.75, *p* < 0.001). The only protective factor identified was phosphate binder use, which afforded a 63% lower fracture risk. As 82% of patients in this study were on calcium-based phosphate binders, it could be speculated that improved phosphate control or the calcium absorption from the binder may have had some protective benefit [48]. Thus, as in healthy young people, the growing skeleton of children with CKD may also need sufficient calcium to promote effective bone mineralization. A similar high prevalence of fractures had been reported in a study of 170 children and young people up to 21 years old in CKD 2–5 and on dialysis. At least one fracture was reported in 6.5% of the children in a one-year follow-up period. Independent risk factors attributed to the fracture risk were rapid growth in adolescence, lower calcium and 25(OH)D levels as well as a higher PTH at the baseline assessment. A lower cortical BMD Z-score predicted future fractures; the hazard ratio for fractures was 1.75 (95% CI 1.15 − 2.67; *p* = 0.009) per standard deviation decrease in baseline BMD [63]. On longitudinal follow-up of this cohort, children with lower serum calcium levels had a loss of BMD, associated with higher PTH levels. The longitudinal change in cortical BMD with the associated increase in calcium levels was most marked in children showing linear growth [63].

These clinical manifestations of bone disease linked to CKD-MBD that develop during the pre-transplant period may be further exacerbated after transplantation. A study following children who received a solid-organ transplant over five years, showed that they had a sixfold higher incidence of fractures overall, but particularly vertebral fractures (160-fold) compared to healthy peers [70].

The dysregulated mineral homeostasis in CKD produces far-reaching consequences even into adulthood, with significant associated morbidity. A study of 249 young adults with childhood onset of CKD stage 5 showed that 37% had symptoms of bone disease (deformities, bone pain, aseptic bone necrosis and atraumatic fractures), 18% were disabled by bone disease and 61% had severe growth restriction [62]. A recent study in children and young adults with CKD or on dialysis has shown that significant daily bone pain occurred in 58% of participants. The most common sites were the lower limbs, back and hips. The pain inhibited activities of daily living and required the frequent use of analgesia. Ten percent of the participants reported at least one previous atraumatic fracture during their time with eGFR < 30 ml/min/1.73 m^2^ [61].

Thus, as in healthy young people, the growing skeleton of children with CKD also needs calcium.

## Mineralization abnormalities on bone histology

Mineralization abnormalities have been shown in many bone biopsy specimens of children with CKD (see Table 2). Bakkaloglu et al. reviewed bone biopsies of 161 children on PD and identified mineralization abnormalities, characterised by increases in both osteoid volume and osteoid maturation time in 48% of all patients. Abnormal mineralization was found in 58% of participants with high bone turnover, 38% with normal turnover and 29% with low turnover. Of note, routinely used clinical markers such as serum PTH and alkaline phosphatase correlated with bone turnover (PTH: r = 0.61, *p* < 0.01; alkaline phosphatase: r = 0.51, *p* < 0.01) but only serum calcium was inversely related to mineralization (*p* < 0.01). In any turnover state, higher PTH values and lower calcium values were associated with abnormal mineralization [59]. In a separate study of 60 children on dialysis, 80% of participants had poor mineralization despite treatment with active vitamin D analogues [71]. Similar results were found in a cohort of 42 children on dialysis with low turnover bone disease in 59%. Abnormal mineralization was found in 29% of them and associated with higher PTH and alkaline phosphatase levels, as well as negatively with serum calcium [72]. Most importantly, mineralization is a significant abnormality in children with CKD [73], and is seen even in the early stages of CKD, before abnormalities in serum calcium, phosphate or PTH manifest, increasing as the eGFR decreases (29% of those in CKD stage 2, 42% in CKD stage 3, and 79% in CKD stages 4/5) [46]. In 60 paediatric patients on peritoneal dialysis, the majority of whom had mineralization defects on bone biopsy, treatment with active vitamin D sterols (calcitriol or doxercalciferol) and phosphate binders (calcium carbonate or sevelamer) for 8 months did not normalise the mineralization indices despite controlled PTH levels [71].

**Table 2:** Mineralization abnormalities found on bone biopsy in children and young adults

Authors, Year (reference)	Population (n) and CKD / dialysis status	Age (years)	Key Findings on bone biopsies	Mineralization abnormalities*
Salusky et al. 1988 [74]	PD (44)	6–18	Normal histology in 16% Osteitis fibrosa in 39% Aplastic lesions in 11% Osteomalacia in 9%	9% of participants Study prior to TMV criteria
Mathias et al. 1993 [75]	HD (21)	16–19	High turnover disease in 38% Osteitis Fibrosa in 23% Adynamic bone in 28%	19% of participants with mixed lesions Study prior to TMV criteria
Goodman et al. 1994 [76]	PD (14)	13–14	*Before calcitriol:* Osteitis Fibrosa in 79% *After calcitriol:* Normal in 43% Adynamic in 43% Osteitis fibrosa in 7% Mixed in 7%	7% of participants Study prior to TMV criteria
Yalçinkaya et al. 2000 [77]	PD (17)	7–20	High turnover disease in 47% Low turnover disease in 29% Mixed in 24%	24% of participants
Ziólkowska et al. 2000 [78]	HD (21), PD (30)	7–15	Adynamic bone disease in 27% Normal bone in 37% Osteomalacia in 2% Hyperparathyroidism in 24% Mixed lesions in 10%	12% of participants
Waller et al. 2008 [73]	Pre-Tx (11)	7–16	Low bone turnover disease in 18% Mixed lesions in 27% Hyperparathyroidism in 36%	81% of participants
Bakkaloglu et al. 2010 [59]	PD (161)	0–20	Low turnover in 4% Normal turnover in 39% High turnover in 57%	Abnormal mineralization in 48%
Wesseling-Perry et al. 2012 [46]	CKD2–5 (52)	2–21	High bone turnover in: 13% with CKD3 29% with CKD 4/5	Defective mineralization in: 29% with CKD2 42% with CKD3 79% with CKD4/5
Bacchetta et al. 2013 [79]	PD (33)	2–21	Patients assigned to treatment with growth hormone vs not At baseline: High turnover in 58% Low turnover in 42%	Mineralization lower in those with low bone turnover (*p* < 0.001) Overall mineralization lag time lower in patients treated with growth hormone (p = 0.03)
Nawrot-Wawrzyniak et al. 2013 [80]	HD (7), PD (11)	3–16	At baseline: Low bone turnover 39% Normal turnover 22%	Mineralization lag time shortened after treatment with growth hormone (*p* < 0.05)
Soeiro et al. 2020 [72]	CKD 5D	0–16	Low turnover in 59% Normal turnover in 24% High turnover in 17%	Defective mineralization in 29%

*Prior to the TMV criteria for reporting bone biopsy results, the assumption was made that both osteitis fibrosa and mixed disease are characterized by increased turnover, but osteitis fibrosa has normal mineralization, whereas mixed disease has abnormal mineralization. Equally, osteomalacia and adynamic disease are states of decreased turnover, with abnormal mineralization in osteomalacia and acellularity in adynamic disease [68]. The mineralization rates in adynamic bone disease are subnormal, but for the purposes of this table, if prior to the TMV criteria, they have not been included in the total percentage of mineralization abnormalities[81]

In contrast, adults on dialysis suffer predominantly from low turnover or adynamic bone disease (ABD) [81, 82]. In the past, it was thought that this was related to overtreatment of secondary hyperparathyroidism with calcium (oral, as phosphate binders, or through the dialysate) and vitamin D analogues [83]. However, recent studies have shown a high prevalence of ABD in treatment-naive patients, in early stages of CKD [84], where conditions that inhibit bone remodelling, such as resistance to the action of PTH, reduced levels of calcitriol, deficiency of sex hormones, diabetes, and uremic toxins such as indoxyl sulfate and sclerostin, are present [85, 86]. Under these circumstances, the high turnover disease would only occur later when serum PTH levels could overcome peripheral resistance to this hormone and other factors that inhibit bone formation [87]. With the progression of CKD, elevated PTH would activate the PTH/PTHrP receptor on osteocytes, suppressing sclerostin and increasing the cellular activity of osteoblasts and osteoclasts, resulting in high bone turnover [88]. Also, mineralization defects are far less common in adults, being observed predominantly in individuals with severe forms of secondary hyperparathyroidism [89–91]. In this situation, bone turnover is greatly increased, and mineralization, a process that requires a certain amount of time, will not be completed at the time when bone resorption is already occurring [88, 89, 91].

The state of hyperparathyroidism in CKD leads to calcium and phosphate release from the bones and the skeleton loses its ability to undertake the normal resorption-formation cycle of remodelling. This leads to an inability to absorb and deposit any excess calcium as hydroxyapatite, during transient episodes of hypercalcaemia. Excess calcium may come from the diet or from calcium-based phosphate binders. This has far-reaching consequences, as vascular calcification is found in a far greater proportion of CKD patients compared to the healthy population. So, could this resorptive state of the bones contribute to the vascular calcification and thus the excessive mortality rates?

## Vascular calcification in CKD

CVD accounts for up to 30% of deaths in children on dialysis [92], and a 1000-fold higher mortality rate in young adults on dialysis compared to their healthy peers [93], with a significant decline in survival with worsening kidney function [94]. Vascular calcification has been causally associated with the higher cardiovascular mortality even in young people and in those with earlier stages of CKD [95–100].

In adults, coronary calcification starts early in CKD and progresses rapidly on dialysis. It is found in as many as 40% of patients in CKD stages 3–4 (GFR 33.0 ± 16.0 mL/min/1.73 m^2^) [101], increasing to 57% of incident hemodialysis patients [102], and up to 83% of patients on maintenance dialysis for a median of 3.6 years [103]. Although most studies showing coronary artery calcification (CAC) in adults included older participants [102, 104–106], this has been shown in pediatric patients on dialysis as well [49, 50, 107]. Children, adolescents and young adults with CKD are shown to have CAC with very different prevalences in different reports. In 39 patients aged up to 30 years old, 35% of patients had evidence of CAC [108]. Young adults with childhood onset CKD had a 92% prevalence of CAC [49]. A more recent cohort study of children and young adults with CKD and on dialysis aged 5 to 30 years old, showed that 10% of the participants had evidence of CAC. Overall, 84% of the cohort had either structural or functional changes associated with vascular calcification. Participants on dialysis had significantly increased measures of arterial stiffness [109].

In fact, once coronary calcification is present, it progresses rapidly [108], and is significantly associated with raised serum calcium and phosphate levels [50, 107, 108, 110–112]. Vessel biopsy studies show that calcification is predominantly seen in the medial layer of the arteries [113], also known as arteriosclerosis or Mönckeberg’s sclerosis [113]. In pre-dialysis patients, calcium accumulation correlates with serum calcium and phosphate levels [114]. With increasing dialysis duration, the vessels exhibit a much higher hydroxyapatite crystal deposition in the tunica media of the artery associated with vascular smooth muscle cell (VSMC) death. This process is strongly associated with high circulating calcium and phosphate levels [115].

Medial calcification in the vessels is not simply a passive ‘dumping’ of hydroxyapatite crystals but an active, cell-mediated process with many similarities to bone mineralization. VSMCs undergo osteochondritic changes with an upregulation of osteoblastic proteins [116]. Due to CKD, the normal calcification inhibiting factors such as fetuin A and OPG are downregulated [117], and VSMCs produce calcifying vesicles that contain hydroxyapatite [113, 118, 119]. Once a nidus of calcification is formed, the VSMC undergoes apoptosis, releasing the hydroxyapatite nanocrystals in matrix vesicles, which go on to form other areas of calcification [120], leading to accelerated calcification in the face of hypercalcaemia and hyperphosphatemia of CKD.

Ongoing arteriosclerosis causes progressive vessel stiffness [121]. Arterial stiffness may cause an increase in cardiac afterload, leading to left ventricular hypertrophy and remodelling [122]. The structural changes, such as carotid intima media thickness (cIMT) increase and subsequent stiffening, begin in early CKD stages and functional abnormalities due to remodelling occur in later stages with progression of CVD [123]. This temporal association of structural changes with functional abnormalities needs to be elucidated further [124]. It may serve as a potential surrogate marker for disease progression and effect monitoring, as well as a treatment target. Increased arterial stiffness, as measured by pulse wave velocity (PWV), has been linked to ischaemic heart disease, stroke and CVD events in a meta-analysis of over 17,000 adults [125].

These data suggest that vascular calcification occurs in all age groups with CKD and is driven by hypercalcemia and dysregulated mineral metabolism. The co-existence of vascular calcification and BMD reduction are shown to be a part of the CKD-MBD spectrum [60], but it was not clear if these are parallel or inter-dependent processes.

## Bone–vascular link in CKD

A link between bone demineralization and vascular calcification has been suggested from clinical studies in adults with CKD. A “calcification paradox” [126] wherein skeletal demineralization is associated with concurrent soft tissue and vascular calcification, has been shown in adults on dialysis. Cejka et al. reported that tibial BMD and bone volume/total volume by high resolution pqCT scan were significantly lower in patients with raised CAC scores (*p* < 0.05) [105]. Chen et al. showed that in 231 adult participants aged 28–75 years (mean age 56 years, 95 on hemodialysis, 55 on peritoneal dialysis and 81 transplanted), those with low vertebral body density (measured on a cardiac CT) had higher CAC scores, and a higher all-cause mortality [127]. Malluche et al. showed that over one year of follow-up of patients on dialysis, three quarters had CAC progression. Progression of CAC was higher in patients with BMD loss (*p* = 0.001). Importantly, with adjustment for age, bone demineralization was a predictor for CAC progression (*β* = 4.6; 95% CI 1.8 to 7.5; *p* = 0.002) [106].

Studies examining coronary calcification in relation to bone biopsy findings have reported that both low and high turnover disease is associated with calcification. Asci et al. performed bone biopsies in 207 adult patients receiving haemodialysis (participants aged 32–75 years). Of these patients, 69% had CAC. Higher CAC scores were associated with increasing age, dialysis vintage, and bone turnover (*p* = 0.013). Low bone turnover was negatively associated with CAC (*p* = 0.03) and high bone turnover was positively associated with CAC (*p* = 0.01) [128]. Barreto et al. have shown in adult dialysis patients that both high and low turnover abnormalities showed calcification progression over one year of follow-up. Conversely, patients with non-progressing calcification, with an initial high turnover subsequently had decreased bone formation rate, and those initially with low turnover subsequently had increased bone formation rate (*p* = 0.003) and osteoid volume [129]. Another study of adult haemodialysis patients measured vascular calcification and bone turnover pre- and post-parathyroidectomy. After surgery, vascular calcification did not progress in the initial 6 months (hungry bone syndrome period). However, as alkaline phosphatase returned to normal levels, coronary calcification score started to increase again [130].

The co-existence and association between bone demineralization and vascular calcification is not only seen in adult dialysis patients but also in pre-dialysis patients. Filgueira et al. found that 50% of 72 adults (age 52 ± 11.7 years, eGFR 40.4 ± 18.2 ml/min/1.73 m^2^) had coronary calcifications (severe calcification in 19%; > 400 Agatston units). Coronary calcifications and vertebral body bone mineral density was inversely correlated (*p* = 0.01), with the highest CAC scores in those within the lowest tertile for BMD (*p* = 0.04) [131].

In addition to the above studies linking vascular calcification to bone demineralization, randomized controlled trials and metanalyses have repeatedly shown a higher all-cause mortality associated with calcium-based phosphate binder use, compared to non-calcium-based binders [132, 133]. This has led to an association between calcium and worse outcomes and a concerted effort to limit calcium intake, through diet or through medications and dialysis fluid, in adult CKD and dialysis patients [134].

Importantly, all the above studies showing CAC association with bone demineralization in adults, and importantly their respective progression, examined participants over the age of 40, with the average age being around 65 years old. As explained in earlier sections of this review, it is crucial that data from studies in older adults are not extrapolated to children or young adults with CKD given the extremes of bone physiology that are seen in the young and old. The growing skeleton of children and young people up to 30 years old is a different metabolic entity, as calcium accrual should still be ongoing. Perhaps this means that the growing skeleton is able to buffer and absorb excess calcium, that is thought to start the vascular calcification cascade in older individuals? Alternatively, due to CKD, perhaps the buffering capacity of bone for calcium is lost and even young people with CKD are at an increased risk of vascular calcification?

There is a paucity of studies examining bone and cardiovascular status in children and young adults with CKD. Preka et al. demonstrated that trabecular thickness by high resolution-pQCT (HR-pQCT) was positively associated with diastolic and mean arterial BP [135]. Ziolkowska et al. showed that cIMT correlated with lumbar spine BMD and total body DXA [136]. Both studies were cross-sectional, so the causal effect of BMD on surrogate markers of vascular calcification cannot be determined.

Our group undertook a multi-centre, longitudinal study, recruiting 100 participants aged 5–30 years old [137]. Twenty-three patients had CKD 4–5 and 75 were on dialysis. The median dialysis duration was 2.5 years (IQR 0.8, 5.1). At baseline, there was significant structural and functional vascular disease, with the medican cIMT z-score raised at 2.2 (IQR 1.1, 2.9) and median PWV z-score at 1.5 (IQR –0.2, 2.6). Ten percent of the young cohort had CAC (Agatston score range 0 to 413). We showed that for 57 patients, over 1.5 years follow-up, surrogate markers of vascular calcification and vessel stiffness (cIMT, PWV) worsened despite ongoing bone mineralization. An annualized increase in trabecular BMD Z-score was an independent predictor of cIMT Z-score increase (R^2^ = 0.48, β = 0.40, *p* = 0.03). However, young people who still demonstrated statural growth, had attenuated vascular changes (lower cIMT, PWV and carotid distensibility annualised Z-score changes) [137]. These results suggested that statural growth may be a significant factor in the relationship between bone and blood vessels in CKD. The process of ongoing bone mineral accrual may be a protective factor, buffering the vasculature from high circulating calcium and phosphate. It may be hypothesized that when the period of rapid growth in adolescence ceases, then the absorption of minerals by the skeleton decreases, leading to increasing structural arterial wall changes.

## Other factors that affect bone demineralization and vascular calcification

Microinflammation seems to be a key factor for both vascular calcification and bone disease in CKD. Studies have shown an association between inflammatory markers, such as IL-6, TNF-α and C-reactive protein and low bone volume [138, 139]. The question that arises is whether inflammation induces bone disease leading to a pro-calcifying milieu, or if calcification comes first, followed by bone loss. A third hypothesis would be inflammation leading simultaneously to bone and arterial disease. In favor of the first hypothesis, ex vivo culture of peripheral blood mononuclear cells of patients undergoing hemodialysis showed spontaneous osteoclastogenesis and release of inflammatory cytokines such as LIGHT, a new pro-osteoclastogenic cytokine, and RANKL [140]. Also, mutations in several macrophage-related genes might affect bone mass [141]. Conversely, the osteoblastic/osteocytic differentiation of VSMCs driven by inflammation could increase the secretion of sclerostin by these cells, inhibiting calcification. The consequence would be a decrease in bone formation rate [142]. In line with this hypothesis, aortas derived from uremic rats that were transplanted into healthy animals caused a decrease in bone mass. These uremic vessels secreted sclerostin in ex vivo cultures, strongly suggesting the role of vascular sclerostin in the bone mass loss [143].

Nevertheless, the bone–vascular axis points to causal factors common to both conditions [126, 144]. Malnutrition-inflammation complex syndrome (MICS) promotes vascular calcification in CKD. A study in uremic rats showed that low dietary protein raised serum levels of osteocalcin and increased vascular calcification [145]. Other studies showed that hypoalbuminemia was associated with low serum fetuin-A [145, 146] and negatively associated with calcification score [147]. On the other hand, the development of vascular calcification and bone disease occurs in parallel with micronutrient deficiencies. Vitamin K-dependent proteins (VKDP), as well as osteocalcin (OC) and matrix Gla-protein (MGP), are essential regulators of bone mineralization [148] which may contribute to greater understanding of the development of vascular calcification, and provide potential therapeutic tools. In CKD patients, vitamin K deficiency was the strongest predictor of vertebral fractures, aortic calcification, and iliac calcification in hemodialysis patients [149]. Moreover, magnesium may inhibit the calcification of VSMCs induced by phosphate. It may suppress the maturation of calciprotein particles (CPPs), which in turn, participate in the pathogenesis of vascular calcification [150]. In CKD patients, magnesium deficiency aggravates inflammation and contributes to vascular calcification and mortality [150]. Furthermore, hypomagnesemia is associated with an increased risk of hip fracture in patients undergoing hemodialysis/hemodiafiltration [151].

Dyslipidemia is an important risk factor for BMD and vascular calcification in CKD patients. Apolipoprotein E is a ligand for receptors that clear chylomicrons and VLDL remnants. CKD-induced ApoE knock-out mice exhibited a significant increase of atherosclerotic plaque in the thoracic aorta, associated with high bone turnover and mineralization defects [152].

Deregulations in gut microbiota also contribute to vascular and bone disease in CKD patients, the “gut–vascular–bone axis”. For example, gut microbiota moving from saccharolytic to proteolytic fermentation pattern, an altered intestinal barrier function, and low vitamin K may promote vascular calcifications [153]. In addition, in CKD patients, gut dysbiosis and increased intestinal permeability contribute to the accumulation of serum uremic toxins, including p-cresyl sulfate (P-CS), indoxyl sulfate (IS), and trimethylamine N-oxide (TMAO), all of which have pro-inflammatory effects [154]. IS induces reactive oxygen species (ROS) generation and stimulates an osteogenic phenotype in VSMCs [155]. Clinical studies demonstrated a positive association between IS serum levels and PWV and aortic calcification in different stages of CKD in adults, [156, 157] and cIMT and progression of PWV in children, independent of other risk factors [158]. A study of osteoblastic cells showed that IS suppresses bone formation, induces osteoclastic bone resorption indirectly by IL-1, and suppresses the differentiation of macrophages into mature osteoclasts, suggesting a direct action of IS in bone tissues [159]. Another in vitro study showed impaired mineralization in MSC-osteoblastic cells treated with P-CS and IS [160].

There is increasing evidence suggesting a close relationship between vascular calcification and bone disease. However, the common pathogenetic mechanisms that involve the bone–vascular axis are still unknown. The elucidation of these mechanisms may contribute to the development of vascular calcification and bone biomarkers and provide potential therapeutic tools.

## Calcium balance studies in CKD

Calcium must play a major role in vascular calcification, and transient hypercalcaemia, either due to dialysis, calcium-based phosphate binders or vitamin D analogues, may influence vascular calcification [32–34]. However, serum calcium levels do not reflect the total body calcium content as 99% of the total body calcium is stored in the bones. In addition, low turnover or adynamic bone cannot buffer any excess calcium or phosphate or transient increases in these, and may further promote or directly cause extraskeletal calcification [34]. Calcium balance studies, with strictly controlled calcium intake and output measurements, that allow calculation of the net calcium gain or loss are extremely challenging and laborious, with only two calcium balance studies in adult CKD patients to date [134, 161]. This is further complicated in dialysis patients where dialysis may induce positive or negative fluxes of calcium in soft tissues and bones.

Healthy adults are generally in a neutral balance; up to the age of 50 years in women and 60–70 years in men, with postmenopausal women and older adults developing physiological bone loss and a negative calcium balance [134]. In patients with CKD, a positive calcium balance may indicate extraskeletal calcification, as opposed to bone deposition. Meta-analyses in adult participants tend to find a higher cardiovascular morbidity and mortality associated with calcium-based phosphate binders [132]. Calcium balance studies in CKD patients have shown that participants on a higher calcium intake were in a positive calcium balance, compared to participants on a low calcium intake. However, their urine calcium output did not differ [161, 162]. The main concern this raises, therefore, is whether this calcium retention reflects extraskeletal calcification.

It is widely accepted that biomarkers such as calcium, phosphate and alkaline phosphatase do not perform well in quantifying bone mineralization in the general population and in disease states such as osteoporosis or CKD. An accurate, non-invasive and easily reproducible biomarker of bone and vascular health is needed in patients with CKD in order to target treatments appropriately. Naturally occurring calcium isotopes and their ratio (δ^44/42^Ca) in the serum and urine seem promising and have so far shown good positive correlation with bone formation markers and negative correlation with bone resorption markers in healthy children, children and young adults with CKD [163] and older women with osteoporosis [164, 165].

Further, detailed calcium balance studies are needed in children and young adults with CKD, where the mineralizing bone requires a higher calcium intake. Calcium may be a protective factor against bone fragility and demineralization as suggested in some clinical studies [63]. In this population, perhaps calcium containing phosphate binders may be preferable to non-calcium containing binders. The need to reconcile this requirement with the burden of vascular calcification needs to be explored in large longitudinal studies in children and young adults, examining the link between bone and vessel calcification in CKD.

## Conclusion

Management of CKD-MBD in the growing child requires the treating physician to reconcile the skeletal requirements for calcium, phosphate and vitamin D, but simultaneously avoiding exposure to excess calcium that can lead to vascular calcification and increase cardiovascular mortality. The growing skeleton may provide a degree of protection by buffering an excess calcium intake, especially during periods of rapid linear growth. The management of CKD-MBD in young adults more closely reflects that in children rather than that seen in older adults on dialysis.

## Summary points

1. Adequate calcium intake is vital for normal bone mineralization in childhood.
2. Healthy people are in a positive calcium balance until the third or fourth decade of life.
3. Young people with CKD can have a significant morbidity from bone disease, manifesting with bone pain, slipped epiphyses, limb deformities or fractures.
4. In children with CKD the predominant finding on bone biopsy is abnormal mineralization.
5. There is increasing evidence that bone demineralization and vascular calcification occur concurrently in children and adults with CKD, and the processes may be linked.
6. Management of CKD-MBD in young adults should follow the principles of treatment in children, rather than of the calcium-sparing strategies followed in older adults.

## Multiple choice questions (answers appear below)

1. Which statement is correct:
  a The process of bone formation, resorption and remodelling is known as bone turnover
  b Cortical bone is more metabolically active than trabecular bone
  c The trabecular compartment is the mineral rich, dense bone compartment
  d There are only 2 cell types in the bone; osteoblasts and osteoclasts
  e The predominant driving force in bone in adults is bone formation
2. Clinical manifestation of mineral bone disease includes:
  a Bone pain
  b Limb deformities
  c Fractures
  d Slipped epiphyses
  e All of the above
3. Calcium balance is:
  a Positive until around 30 years of age
  b Positive until around 15 years of age
  c Positive until around 50 years of age
  d Negative after 60 years of age
  e Neutral throughout life
4. The predominant abnormality found in bone biopsies of children with CKD is:
  a Low bone turnover
  b High bone turnover
  c Abnormal mineralization
  d Osteitis fibrosa
  e Aluminium staining
5. Which statement is correct:
  a Vascular calcification is associated with an increased morbidity and mortality in people with CKD
  b Coronary artery calcification is only seen in older adults with CKD
  c Dialysis attenuates the progression of vascular calcification
  d Vascular calcification involves dumping of excess calcium and phosphate from calcium-containing medications in blood vessels

### Supplementary Information

Below is the link to the electronic supplementary material.

Graphical abstract (PPTX 62 KB)
