# Supplementary material for: Chronic kidney disease mineral bone disorder in childhood and young adulthood: a ‘growing’ understanding
Source: Pediatr Nephrol. 2023 Aug 25;39(3):723–39. doi: 10.1007/s00467-023-06109-3 (PMC10817832; doi:10.1007/s00467-023-06109-3)
Supplement: Supplementary file 1 — Graphical abstract (PPTX 62 KB) [file 467_2023_6109_MOESM1_ESM.pptx]

## Slide 1
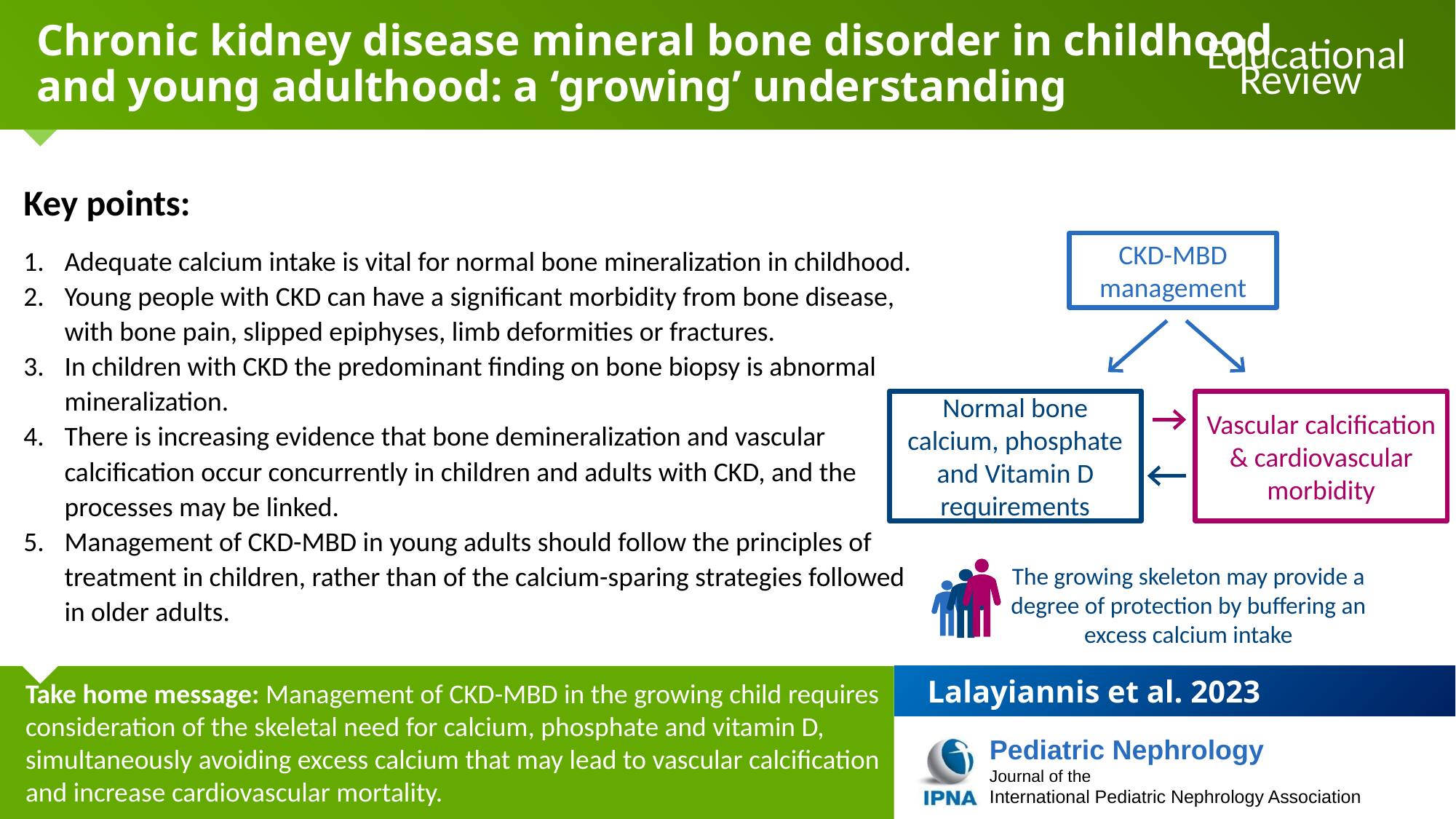

Chronic kidney disease mineral bone disorder in childhood
and young adulthood: a ‘growing’ understanding
Key points:
Adequate calcium intake is vital for normal bone mineralization in childhood.
Young people with CKD can have a significant morbidity from bone disease, with bone pain, slipped epiphyses, limb deformities or fractures.
In children with CKD the predominant finding on bone biopsy is abnormal mineralization.
There is increasing evidence that bone demineralization and vascular calcification occur concurrently in children and adults with CKD, and the processes may be linked.
Management of CKD-MBD in young adults should follow the principles of treatment in children, rather than of the calcium-sparing strategies followed in older adults.
CKD-MBD management
Vascular calcification & cardiovascular morbidity
Normal bone calcium, phosphate and Vitamin D requirements
The growing skeleton may provide a degree of protection by buffering an excess calcium intake
Lalayiannis et al. 2023
Take home message: Management of CKD-MBD in the growing child requires consideration of the skeletal need for calcium, phosphate and vitamin D, simultaneously avoiding excess calcium that may lead to vascular calcification and increase cardiovascular mortality.
